# Creating an arsenal of Adeno-associated virus (AAV) gene delivery stealth vehicles

**DOI:** 10.1371/journal.ppat.1006929

**Published:** 2018-05-03

**Authors:** J. Kennon Smith, Mavis Agbandje-McKenna

**Affiliations:** Department of Biochemistry and Molecular Biology and Center for Structural Biology, the McKnight Brain Institute, University of Florida, Gainesville, Florida, United States of America; University of Kentucky, UNITED STATES

## Abstract

The Adeno-associated virus (AAV) gene delivery system is ushering in a new and exciting era in the United States; following the first approved gene therapy (Glybera) in Europe, the FDA has approved a second therapy, Luxturna [1]. However, challenges to this system remain. In viral gene therapy, the surface of the capsid is an important determinant of tissue tropism, impacts gene transfer efficiency, and is targeted by the human immune system. Preexisting immunity is a significant challenge to this approach, and the ability to visualize areas of antibody binding (“footprints”) can inform efforts to improve the efficacy of viral vectors. Atomic resolution, smaller proteins, and asymmetric structures are the goals to attain in cryo-electron microscopy and image reconstruction (cryo-EM) as of late. The versatility of the technique and the ability to vitrify a wide range of heterogeneous molecules in solution allow structural biologists to characterize a variety of protein–DNA and protein–protein interactions at lower resolution. Cryo-EM has served as an important means to study key surface areas of the AAV gene delivery vehicle—specifically, those involved with binding neutralizing antibodies (NAbs) [2–4]. This method offers a unique opportunity for visualizing antibody binding “hotspots” on the surface of these and other viral vectors. When combined with mutagenesis, one can eliminate these hotspots to create viral vectors with the ability to avoid preexisting host immune recognition during gene delivery and genetic defect correction in disease treatment. Here, we discuss the use of structure-guided site-directed mutagenesis and directed evolution to create “stealth” AAV vectors with modified surface amino acid sequences that allow NAb avoidance while maintaining natural capsid functions or gaining desired novel tropisms.

## Q1: What properties of the AAV capsid surface assist or hinder gene delivery?

**Fig 1:**
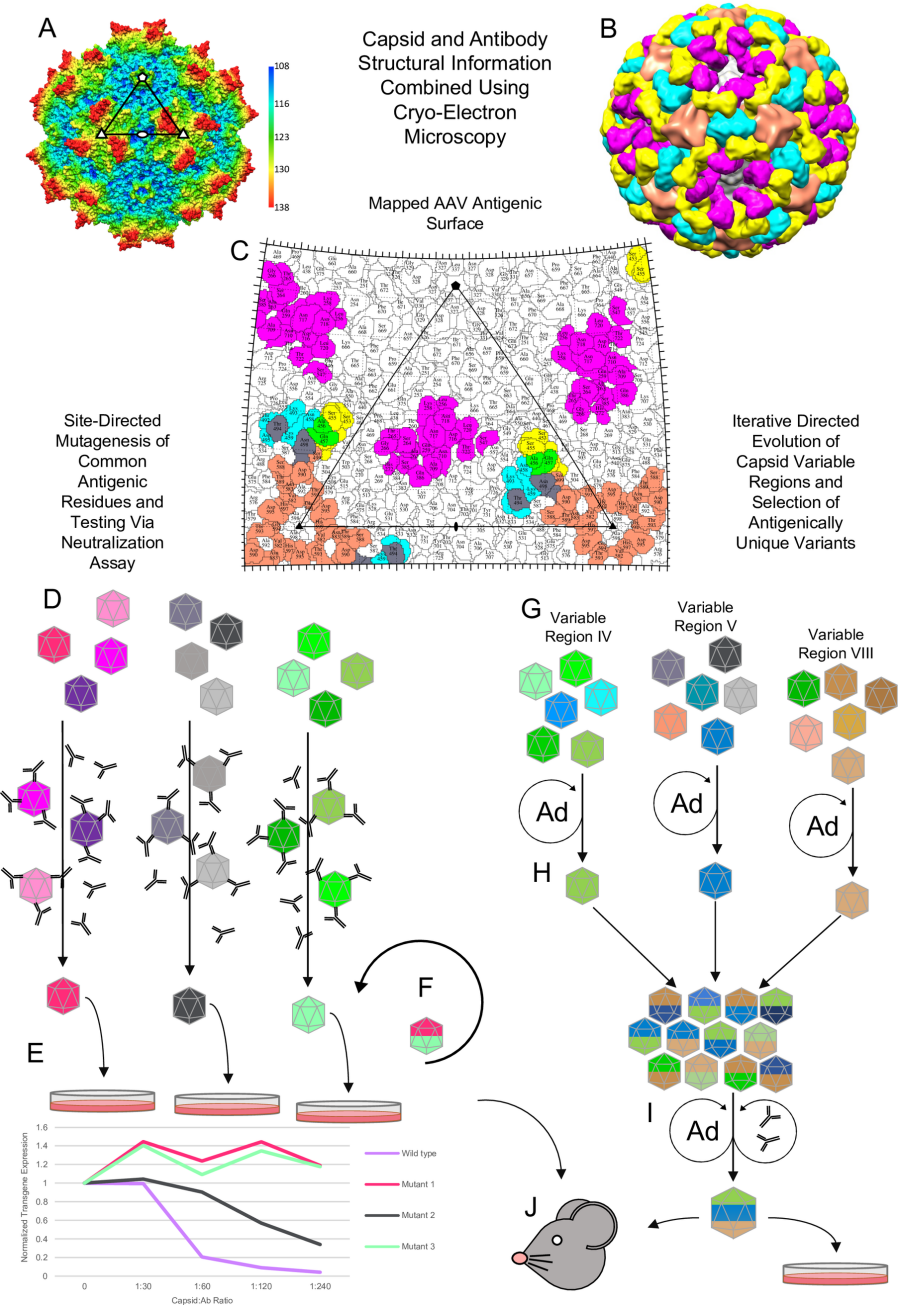
Steps to creating stealth AAV vectors. (A) AAV1 capsid structure. The view is along the two-fold axis. The image is radially colored, as indicated in the color key. The viral asymmetric unit is indicated by the triangle joining 2-, 3-, and 5-fold axes indicated by filled oval, triangles, and pentagon, respectively. The raised surface between the 2- and 5-fold axes is the 2/5-fold wall. (B) AAV1 capsid decorated with FAbs from 4 different anti-AAV1 MAbs: 5H7 (orange), 4E4 (cyan), ADK1a (yellow), and ADK1b (magenta). The view is as in (A). (C) 2D surface roadmap [7] of AAV1 showing the footprints from the FAbs in (B). Roadmaps allow for the visualization of surface-accessible residues within a capsid viral asymmetric unit. Here, residues important for antibody interactions with the AAV1 capsid are highlighted. Asymmetric unit and axis of symmetry are indicated as in (A) and (B). (D) Site-directed mutagenesis of CAMs as colored. Individual epitopes are mutated. (E) Neutralization assays (with rAAV vectors) to test escape phenotype in permissive cells lines. Assembled mutant capsids are challenged with a panel of MAbs, IVIG, or an individual serum. (F) Iterative mutation testing. Variants showing transduction comparable to wild-type virus, as well as NAb escape, are combined for additional testing to maximize effect. Epitopes remaining recalcitrant to escape are reengineered in the background of escaping vectors. (G) Synthetic peptides are substituted for individual CAMs (as colored), and capsids are selected through several rounds of iterative evolution in the presence of Ad helper virus. (H) Combination of CAM site variants after the first round of selection (as colored). (I) Additional round of selection done in the presence of MAbs and Ad helper. (J) rAAV vectors, made using either method, are tested in vivo for their ability to transduce target tissues in comparison to wild-type virus with or without MAbs, IVIG, or individual serum added as pressure. AAV, Adeno-associated virus; AAV1, Adeno-associated virus serotype 1; Ab, antibody; Ad, Adenovirus; CAMs, capsid antigenic motifs; FAbs, fragment antibodies; IVIG, intravenous immunoglobulin; MAbs, monoclonal antibodies; rAAV, recombinant Adeno-associated virus.

The AAVs, approximately 260 Å in capsid diameter, are nonpathogenic and helper-dependent parvoviruses widely utilized for gene delivery applications, both at the bench and in clinical trials (ClinicalTrials.gov). They have been co-opted to deliver treatments for congenital genetic diseases, clustered regularly interspaced short palindromic repeats (CRISPR)/CRISPR-associated protein (Cas)–driven in vivo gene editing, and opsin delivery in optogenetics. Efforts to improve gene delivery efficacy often include tissue-specific targeting and increasing trafficking efficiency via manipulation of the capsid surface. Structural biologists think of the AAV capsid surface as having distinct regions defined by 60 viral proteins whose interfaces form icosahedral 5-, 3-, and 2-fold symmetry–related interactions in a T = 1 capsid (Fig 1A). The interactions at these axes juxtapose functional regions on the capsid surface required for the various steps that enable successful gene delivery [5]. For example, the 3-fold axis and its surroundings contain cellular receptor attachment sites, and a channel at the 5-fold axis is proposed to function to externalize an essential phospholipase A2 enzyme required for endo- and lysosomal trafficking post-entry [6]. Equally important are regions facilitating antibody recognition that hinder infection. Epitope mapping using different techniques—including random mutagenesis, peptide insertion, and cryo-EM of capsid–antibody complexes—has shown that regions required for viral function are often targeted by antibodies, and a polyclonal reaction would leave much of the surface covered and unavailable for cellular function (Fig 1B). These include the 2/5-fold wall, the 3-fold region, and around the 5-fold axes.

## Q2: How does preexisting immunity limit AAV vector gene delivery?

Although there exist numerous genetic isolates and recombinant variants of AAV [8, 9], the detrimental effects of antibody binding to AAV gene delivery in preclinical and clinical studies are well documented [10, 11]. Natural exposure to AAV capsids results in 40%–70% seropositivity in the general population, and preexisting immunity is an exclusion criterion for clinical trials [12]. While not all the antibodies in human serum are neutralizing, NAbs can disrupt the multiple steps required for transgene delivery, from cellular attachment to post-entry trafficking and capsid uncoating events, and can greatly reduce transduction efficiency or alter biodistribution [2, 13–16]. Cross-reactivity among serotypes, due to regions of sequence and structure conservation, serves as an additional hindrance to treatment, further exacerbating the problem [12, 17]. Strategies to subvert the host immune system to enable efficient gene delivery include the use of capsid “decoys” in the form of empty (no DNA) viral capsids to “soak up” antibodies and immunosuppression [18]. Recently, an immunosuppression regimen using a combination of rapamycin and prednisolone was effective at depleting preexisting anti-AAV serotype 9 antibody development and production in a mouse model [19]. An attractive alternative, given that immunosuppression is a short-term solution, is to use information on dominant capsid antigenic motifs (CAMs), conserved among autonomous parvoviruses and the AAVs [3, 20], to develop antibody escape vectors. AAV escape variants can be created by random site-directed mutagenesis, error prone PCR, directed evolution, and structure-guided rational site-directed or structure-guided rational directed evolution.

## Q3: How does one use cryo-EM to map antigenic footprints?

Single-particle cryo-EM is experiencing a resolution revolution in structure biology, facilitated by advancements in detector technology with novel data collection and processing software development [21]. To create an antigenic “map” of each AAV serotype using cryo-EM, first a panel of antibodies is generated against the capsid in an effort to recapitulate a polyclonal response. Purified fragment antibodies (FAbs) and the AAV capsids are mixed to create virus–antibody complexes for data collection. Symmetric disposition of FAbs on the capsids aids data processing and cryo-EM (Fig 1B), although 60 copies of FAbs are not required to visualize binding sites [22]. Interpretation of the capsid–FAb complex structures has involved the computational docking of available 3D structures of the capsid and a generic FAb structure into the reconstructed density map [2, 3, 14]. However, advances in this approach now result in atomic resolution information for complex structures. Points of direct amino acid interaction between the capsid and FAb, as well as capsid residues that are occluded by antibody binding, form each footprint. Multiple capsid–antibody structures are amassed to visualize “polyclonal” contacts on each capsid surface. For capsid engineering purposes, structural alignment of the footprint residues mapped for different AAV serotypes for multiple antibodies has been conducted and shows commonality on the AAV capsid localized to 3 regions: the 2/5-fold wall, the 3-fold protrusions, and surrounding the 5-fold axes (Fig 1A) [3]. CAMs identified on multiple serotypes allow the use of similar mutagenesis strategies to target the modification of their footprints to specific residues when creating host immune escape vectors. A 2D roadmap can be used to depict the footprint residues mapped by cryo-EM and localized to the capsid surface (Fig 1C).

## Q4: How are mapped footprints applied to escape vector engineering?

NAbs work by blocking or competing with important functions required for virus infection, so care must be taken to preserve these functions when eliminating their binding [14]. To knock out polyclonal recognition, site-directed mutagenesis or directed evolution of CAM footprint residues can be used (Fig 1D or 1G). In the first method, one or few residues are changed on the capsid surface in order to maintain the parental infection properties, while introducing an escape phenotype [2] (Fig 1D and 1F). The ability of these recombinant AAVs (rAAVs) to escape from monoclonal antibodies (against which they were developed) as well as polyclonal samples—such as intravenous immunoglobulin (IVIG) and individual human sera—are then tested in vitro and in vivo, in comparison to their parent capsid (Fig 1E, 1F and 1J). In the second method, stretches of residues within the structurally mapped footprint are altered with small peptide substitutions containing a combination of all amino acid types at each residue position, generating a library of surface loop variants. Sequences that are able to assemble capsids and retain or improve their transduction capabilities are selected with Adenovirus (Ad) coinfection alone or in the presence of Ad and antibodies as described above, in specific tissues or cells (Fig 1G–1I). In both approaches, the ability of the arising vectors to transduce target tissues can also be tested without or with NAbs in mice or nonhuman primates. A recent directed evolution study using AAV1 generated capsid libraries with variations at CAMs localized to the 3-fold protrusions (Fig 1G, 1H and 1J) and selected novel vectors in vascular endothelial cells [4]. Although this approach can drastically change the wild-type capsid sequence, selection in specific cells inherently checks against mutations that negate vector performance while ensuring stealth vehicle creation.

## Summary

Preexisting host immunity remains an obstacle to full realization of the AAV gene delivery system due to its negative effect on transgene expression. Cryo-EM of AAV–antibody complexes, combined with methods to alter structurally mapped antigenic footprints, provides a powerful approach for overcoming the humoral response against the capsid. Recapitulating the polyclonal response by mapping multiple monoclonal epitopes can aid the development of vectors with broad immunogenic escape. In the future, libraries of antigenic variants will allow for repeat administration, creating a vector development strategy complementary to current clinical methods of maximizing therapeutic gene delivery, such as plasmapheresis or B cell suppression. The results thus far highlight the positive impact that structure-based approaches can have on engineering gene therapy vectors with improved efficacy and greater patient participation among those with preexisting immunity.

## References

[1] FDA approves novel gene therapy to treat patients with a rare form of inherited vision loss. [Internet]. FDA.gov. 2017 Dec 19 [cited 09 April 2018] Available from: https://www.fda.gov/NewsEvents/Newsroom/PressAnnouncements/ucm589467.htm.

[2] Gurda BL, Raupp C, Popa-Wagner R, Naumer M, Olson NH, Ng R, et al Mapping a Neutralizing Epitope onto the Capsid of Adeno-Associated Virus Serotype 8. J Virol. 862012. p. 7739–51. 22593150 10.1128/JVI.00218-12 PMC3421660

[3] Tseng YS, Gurda BL, Chipman P, McKenna R, Afione S, Chiorini JA, et al Adeno-associated virus serotype 1 (AAV1)- and AAV5-antibody complex structures reveal evolutionary commonalities in parvovirus antigenic reactivity. J Virol. 2015;89(3):1794–808. Epub 2014/11/21. doi: 10.1128/JVI.02710-14 ; PubMed Central PMCID: PMCPMC4300747. 25410874 10.1128/JVI.02710-14 PMC4300747

[4] Tse LV, Klinc KA, Madigan VJ, Rivera RMC, Wells LF, Havlik LP, et al Structure-guided evolution of antigenically distinct adeno-associated virus variants for immune evasion. 2017 doi: 10.1073/pnas.1704766114 28559317 10.1073/pnas.1704766114 PMC5474820

[5] Agbandje-McKenna M, Kleinschmidt J. AAV capsid structure and cell interactions. Methods Mol Biol. 2011;807:47–92. doi: 10.1007/978-1-61779-370-7_3 . 22034026 10.1007/978-1-61779-370-7_3

[6] Huang LY, Halder S, Agbandje-McKenna M. Parvovirus glycan interactions. Curr Opin Virol. 2014;7:108–18. doi: 10.1016/j.coviro.2014.05.007 ; PubMed Central PMCID: PMCPMC4149944. 25047752 10.1016/j.coviro.2014.05.007 PMC4149944

[7] Xiao C, Rossmann MG. Interpretation of electron density with stereographic roadmap projections. J Struct Biol. 2007;158(2):182–7. Epub 2006/11/23. doi: 10.1016/j.jsb.2006.10.013 ; PubMed Central PMCID: PMCPMC1978246. 17116403 10.1016/j.jsb.2006.10.013 PMC1978246

[8] Gao G, Vandenberghe LH, Alvira MR, Lu Y, Calcedo R, Zhou X, et al Clades of Adeno-Associated Viruses Are Widely Disseminated in Human Tissues. J Virol. 782004. p. 6381–8. 15163731 10.1128/JVI.78.12.6381-6388.2004 PMC416542

[9] Gao G-P, Alvira MR, Wang L, Calcedo R, Johnston J, Wilson JM. Novel adeno-associated viruses from rhesus monkeys as vectors for human gene therapy. Proc Natl Acad Sci. 2002;99:11854–9. doi: 10.1073/pnas.182412299 12192090 10.1073/pnas.182412299 PMC129358

[10] Boutin S, Monteilhet V, Veron P, Leborgne C, Benveniste O, Montus MF, et al Prevalence of serum IgG and neutralizing factors against adeno-associated virus (AAV) types 1, 2, 5, 6, 8, and 9 in the healthy population: implications for gene therapy using AAV vectors. Hum Gene Ther. 2010;21(6):704–12. Epub 2010/01/26. doi: 10.1089/hum.2009.182 . 20095819 10.1089/hum.2009.182

[11] Manno CS, Pierce GF, Arruda VR, Glader B, Ragni M, Rasko JJ, et al Successful transduction of liver in hemophilia by AAV-Factor IX and limitations imposed by the host immune response. Nat Med. 2006;12(3):342–7. Epub 2006/02/14. doi: 10.1038/nm1358 . 16474400 10.1038/nm1358

[12] Greenberg B, Butler J, Felker GM, Ponikowski P, Voors AA, Pogoda JM, et al Prevalence of AAV1 neutralizing antibodies and consequences for a clinical trial of gene transfer for advanced heart failure. Gene Ther. 2016;23(3):313–9. Epub 2015/12/25. doi: 10.1038/gt.2015.109 . 26699914 10.1038/gt.2015.109 PMC6558655

[13] Harbison CE, Weichert WS, Gurda BL, Chiorini JA, Agbandje-McKenna M, Parrish CR. Examining the cross-reactivity and neutralization mechanisms of a panel of mAbs against adeno-associated virus serotypes 1 and 5. J Gen Virol. 2012;93(Pt 2):347–55. Epub 2011/11/11. doi: 10.1099/vir.0.035113-0 ; PubMed Central PMCID: PMCPMC3352341. 22071509 10.1099/vir.0.035113-0 PMC3352341

[14] Tseng YS, Agbandje-McKenna M. Mapping the AAV Capsid Host Antibody Response toward the Development of Second Generation Gene Delivery Vectors. Front Immunol. 2014;5:9 Epub 2014/02/14. doi: 10.3389/fimmu.2014.00009 ; PubMed Central PMCID: PMCPMC3906578. 24523720 10.3389/fimmu.2014.00009 PMC3906578

[15] Hurlbut GD, Ziegler RJ, Nietupski JB, Foley JW, Woodworth LA, Meyers E, et al Preexisting immunity and low expression in primates highlight translational challenges for liver-directed AAV8-mediated gene therapy. Mol Ther. 2010;18(11):1983–94. doi: 10.1038/mt.2010.175 ; PubMed Central PMCID: PMCPMC2990518. 20736932 10.1038/mt.2010.175 PMC2990518

[16] Li C, Narkbunnam N, Samulski RJ, Asokan A, Hu G, Jacobson LJ, et al Neutralizing antibodies against adeno-associated virus examined prospectively in pediatric patients with hemophilia. Gene Ther. 2012;19(3):288–94. Epub 2011/06/24. doi: 10.1038/gt.2011.90 . 21697954 10.1038/gt.2011.90

[17] Tseng YS, Gurda BL, Chipman P, McKenna R, Afione S, Chiorini JA, et al Adeno-associated virus serotype 1 (AAV1)- and AAV5-antibody complex structures reveal evolutionary commonalities in parvovirus antigenic reactivity. J Virol. 2015;89(3):1794–808. doi: 10.1016/j.jviromet.2016.07.009 25410874 10.1128/JVI.02710-14 PMC4300747

[18] Tse LV, Moller-Tank S, Asokan A. Strategies to circumvent humoral immunity to adeno-associated viral vectors. Expert Opin Biol Ther. 2015;15(6):845–55. doi: 10.1517/14712598.2015.1035645 ; PubMed Central PMCID: PMC4689135. 25985812 10.1517/14712598.2015.1035645 PMC4689135

[19] Velazquez VM, Meadows AS, Pineda RJ, Camboni M, McCarty DM, Fu H. Effective Depletion of Pre-existing Anti-AAV Antibodies Requires Broad Immune Targeting. Mol Ther Methods Clin Dev. 42017. p. 159–68. 10.1016/j.omtm.2017.01.003 PMC5363314 28345001

[20] Hafenstein S, Bowman VD, Sun T, Nelson CD, Palermo LM, Chipman PR, et al Structural comparison of different antibodies interacting with parvovirus capsids. J Virol. 2009;83(11):5556–66. Epub 2009/03/27. doi: 10.1128/JVI.02532-08 ; PubMed Central PMCID: PMCPMC2681957. 19321620 10.1128/JVI.02532-08 PMC2681957

[21] Cheng Y, Grigorieff N, Penczek PA, Walz T. A Primer to Single-Particle Cryo-Electron Microscopy. Cell. 2015;161(3):438–49. doi: 10.1016/j.cell.2015.03.050 ; PubMed Central PMCID: PMC4409659. 25910204 10.1016/j.cell.2015.03.050 PMC4409659

[22] Gurda BL, DiMattia MA, Miller EB, Bennett A, McKenna R, Weichert WS, et al Capsid antibodies to different adeno-associated virus serotypes bind common regions. J Virol. 2013;87(16):9111–24. doi: 10.1128/JVI.00622-13 ; PubMed Central PMCID: PMCPMC3754064. 23760240 10.1128/JVI.00622-13 PMC3754064

